# MRI features and radiomics of the pineal gland in girls do not show significant changes at onset of puberty

**DOI:** 10.3389/fendo.2026.1771500

**Published:** 2026-05-29

**Authors:** Anna-Mariia Shulhai, Michele Maddalo, Maddalena Petraroli, Francesca Ormitti, Benedetta Piccolo, Emanuela Claudia Turco, Nicola Sverzellati, Caterina Ghetti, Susanna Esposito, Maria Elisabeth Street

**Affiliations:** 1 Department of Medicine and Surgery, University of Parma, Parma, Italy; 2 Medical Physics Unit, University Hospital of Parma, Parma, Italy; 3 Unit of Paediatrics, University Hospital of Parma, Parma, Italy; 4 Neuroradiology Unit, University Hospital of Parma, Parma, Italy; 5 Unit of Radiological Sciences, University Hospital of Parma, University of Parma, Parma, Italy

**Keywords:** central precocious puberty, machine learning, magnetic resonance imaging, pineal gland, puberty, radiomics

## Abstract

**Introduction:**

The gold standard for diagnosing central precocious puberty(CPP) is the gonadotropin-releasing hormone stimulation test, along with magnetic resonance imaging(MRI) of the brain and hypothalamus-pituitary region to rule out central organic causes. Recent advancements have led to a new medical imaging approach called radiomics. Our recent study showed that pituitary gland radiomics is a promising tool for diagnosing CPP. However, the role of the pineal gland in the onset of puberty has long been debated. Therefore, we investigated radiomic features of the pineal gland associated with puberty onset to identify changes that could assist physicians in the diagnostic workup of CPP.

**Methods:**

45 girls with a confirmed diagnosis of CPP and 47 pre-pubertal, age-and sex-matched subjects(controls) were retrospectively enrolled. Two readers(R1, R2) with different levels of expertise in pediatric neuroradiology blindly segmented the pineal gland on MRI studies for radiomic features(RFs) calculation and manually evaluated the number and diameter of pineal cysts. Cross-validated linear discriminant analysis was used to develop, for each reader, both a radiomic model and a reference model based on pineal cyst features. Radiomics was evaluated in terms of predictive performances(ROC-AUC) and reliability of predictors between readers (intraclass correlation coefficient). Finally, the correlation between cysts' features and basal/peak gonadotropin and estradiol levels was also investigated.

**Results:**

Two radiomic features were identified as the most predictive of CPP for both readers. However, these features were not the same for R1 and R2 readers and their values showed poor inter-reader reliability. Unpromising performance in the validation set was observed for pineal gland radiomics (ROC-AUC of 0.64 for R1 and 0.59 for R2). Similarly, the reference model based on pineal cyst features demonstrated a poor performance (ROC-AUC = 0.52, both readers). No significant correlations between cyst features and basal/peak gonadotropin levels were observed.

**Conclusion:**

Radiomic features of the pineal gland in girls did not show consistent and relevant changes with the onset of puberty and do not hold promise for the CPP diagnosis at variance with previous findings in the pituitary gland. Similarly, the number and size of cysts were not found to be specific for the onset of puberty.

## 1 Introduction

Central precocious puberty (CPP) is characterized by early activation of the hypothalamus-pituitary-gonadal (HPG) axis (1). This process begins with the secretion of gonadotropin-releasing hormone (GnRH) by the hypothalamus, which stimulates the anterior pituitary to release luteinizing hormone (LH) and follicle-stimulating hormone (FSH). Then the gonadotropins stimulate production of sex steroids by the gonads (2, 3).

The diagnosis of CPP is based on presence of clinical pubertal signs, a GnRH stimulation test, which is yet considered the «gold standard» for diagnosis and additionally, instrumental assessments (4–6). In girls, pelvic ultrasound can confirm estrogenic stimulation of the uterus and ovaries and help exclude cystic or malignant lesions (7). A brain magnetic resonance imaging (MRI) is recommended to exclude underlying central nervous system anomalies (8, 9). Current guidelines suggest that MRI should be performed in all girls under 8 years and boys under 9 years with CPP (8, 9). However, according to the recent literature there is a relatively low probability of detecting pathological findings in girls over 6 years of age (10–13), and most cases of CPP are idiopathic. Finally, bone age, assessed using an X-ray of the non-dominant hand and wrist will be advanced compared to chronological age in CPP (4).

The pineal gland is a small endocrine organ located in the posterior cranial fossa (14). This gland plays a central role in circadian regulation and has been hypothesized to influence pubertal timing via melatonin secretion (15–17). Melatonin is a neuroendocrine hormone that plays a role in regulating biological rhythms, immune function and aging. It can also inhibit the HPG axis by down-regulating the Kiss-1/Kiss1R system (18), and its decline has been hypothesized as one of the factors that may trigger the onset of puberty (18–20). Pineal cysts are a common finding in brain MRIs. They usually represent an incidental and benign finding, however, their high prevalence has raised questions on their potential functional significance (19, 21–23).

The development of artificial intelligence (AI) and its application in medical imaging, i.e. radiomics, has emerged as a promising field capable of extracting quantitative features from standard imaging modalities such as MRI (24, 25). Radiomics aims to detect subtle tissue characteristics that are imperceptible to the human eye and to associate these with clinical, biological and prognostic evaluations, therefore, helping to create a more personalized approach to diagnosis and treatment (26, 27). Deep learning methods, particularly convolutional neural networks (CNNs), have enhanced the performance of image segmentation and feature extraction in neuroimaging (28), and can be used in medical practice. Promising results demonstrated the usefulness of radiomics of the pituitary gland (29–31), due to its dynamic changes in shape and volume during puberty for the diagnosis of CPP, however, the role of other neuroanatomical structures or presence of other congenital features, such as changes in the pineal gland, remains not fully understood. Observational studies have reported controversial results on associations between pineal cysts and onset of puberty prompting for further research in this area (20, 22).

The aim of this study was to investigate the features of the pineal gland in relationship to the onset of puberty, using a radiomics approach in addition to routine imaging, to assess whether there were any specific changes that could assist physicians in the diagnostic workup of CPP.

## 2 Materials and methods

### 2.1 Study design and subjects

Forty-five girls with a confirmed diagnosis of CPP were retrospectively enrolled in the Pediatric Endocrinology Unit at the Children’s University Hospital in Parma, Italy, between January 2015 and December 2022 (29). Forty-seven prepubertal age-matched girls, referred to the Pediatric Neurology Unit of the same hospital that had negative neuroradiological findings on brain MRI scans for non-specific neurological indications (e.g., headache, minor brain trauma, epilepsy) and found to have negative neuroradiological findings on brain MRI, were selected as a comparison group. This subgroup was intentionally sampled to obtain a control cohort with a sample size comparable to that of the CPP group within the same recruitment period.

Inclusion criteria were: females, confirmed CPP diagnosis, availability of MRI data for pineal gland evaluation, obtained informed consent. Exclusion criteria included: male sex, premature thelarche, non-idiopathic CPP, evidence of hypothalamic-pituitary malformations, neurological conditions, intracranial tumors, or other endocrine or chronic diseases, known endocrine disorders requiring treatment, significant comorbidities, or use of any medications potentially affecting pubertal development.

At the first assessment, all patients underwent clinical and auxological evaluation. Pubertal stages were classified according to Tanner’s criteria (32). Height was measured using a Harpenden stadiometer, and body mass index (BMI) was calculated as weight (kg)/height^2^ (m^2^) and reported as standard deviation scores (SDS) using Italian reference standards (33). Target height (THt) was estimated from parental height [father’s height + mother’s height – 13]/2, then converted to SDS (34), and plotted on national growth charts (35).

Medical history and family history of precocious puberty, parents’ height, and age at pubertal onset were recorded. Pubertal progression rate was defined as the time between the onset of Tanner stage B2 (as recorded by the family pediatrician or reported by parents) and the time of diagnosis. Bone age (BA) assessment using radiographs of the non-dominant hand using the Greulich and Pyle method (36). Bone age advancement was defined by the difference between bone age and chronological age.

All girls underwent pelvic trans-abdominal ultrasound to evaluate the degree of internal genitalia maturation. Uterine longitudinal diameter ≥ 35mm, a body/cervix ratio ≥ 1, ovarian volume ≥ 2 mL, and the presence of endometrial thickening were considered consistent with pubertal activation (37).

A GnRH stimulation test was performed via intravenous administration of 75 μg/m^2^ of GnRH (maximum 100 μg), with serum LH and FSH measured at baseline and at +15, +30, +45, +60, and +90 minutes using chemiluminescent immunoassays (Beckman Coulter). Peak LH above 5 IU/L and a LH/FSH peak ratio of > 1.0 were considered diagnostic of CPP (4). In one case, the GnRH test was not performed due to a significant increase in basal gonadotropin levels. All subjects also had their estradiol levels and thyroid function tests assessed, with no abnormal findings. Central precocious puberty was defined based on age, pubertal stages, LH and FSH responses to the GnRH tests, pelvic ultrasound, and bone age (4).

Brain magnetic resonance imaging (MRI) of the hypothalamus, pituitary gland, and pineal gland was performed in all CPP patients.

### 2.2 Ethics statement

This research protocol was approved by the local Ethical Committee (Aven: vast area North Emilia N. Prot. n. 19557 del 09/05/2023). For all participants, informed parental consent was obtained from both parents/legal guardians and from those aged ≥6 years to use the medical data.

### 2.3 Magnetic resonance imaging of the pineal gland

#### 2.3.1 Brain magnetic resonance imaging sequence and delineation of the pineal gland region

All girls diagnosed with CPP underwent brain MRI using axial T2-weighted imaging (T2WI), sagittal 3DT1-weighted imaging (CE-T1WI 3D TFE) with contrast enhancement, and dedicated axial T2-weighted imaging (T2WI 3D DRIVE) at the level of the pineal gland region. MRI was acquired in the head-first supine position on a 1.5-T Philips Ingenia system (Best, The Netherlands) scanner using the 8HRBRAIN radiofrequency coil. Bias-field correction and intensity normalization were not performed during MRI scanning.

The acquisition parameters of T2WI sequence were as follows: acquisition matrix of 364x230, field of view (FOV): 230x184 mm, slice thickness of 4mm, spacing between slices of 1mm, flip angle: 90. Meanwhile, the acquisition parameters of T1WI 3D TFE sequence were as follows: acquisition matrix of 208x194, FOV: 227x213 mm, slice thickness of 1.1mm, spacing between slices of 0mm, flip angle: 8. CE-T1WI 3D TFE was carried out the T1WI sequence parameters after rapid injection of a gadolinium-DTPA contrast agent (0.1 mmol/kg Dotarem). Moreover, dedicated axial T2-weighted imaging (T2WI 3D DRIVE) at the level of the pineal gland region parameters were as follows: trueFiSP TR1500/TE173, FOV:160x160 mm, matrix 268x268, slice thickness 600 µm, Voxel size isotropic (0.6x0.6x0.6 µm). T2WI 3D DRIVE in the axial plane and CE-T1WI in the sagittal plane have been utilized, and all DICOM format images were collected based on the picture archiving and communication system at the Parma University Hospital.

A neuroradiologist with four years of experience (R1) in pediatric pathology and a radiologist with one year of training (R2) conducted manual segmentation of the pineal volume independently and in a blinded fashion using a 3D Slicer (v.5.0.3) (38).

The normal pineal gland is an ovoid structure, less than a centimeter in size along its major axis. In the absence of fluid, its signal intensity on T1- and T2-weighted images is close to that of grey matter. We defined a cyst as any well-demarcated, smooth-edged, fluid-filled formation within the pineal gland that was greater than or equal to 5mm in size in at least one dimension. The pineal borders were manually defined on transversal 3D images (800 µm isotropic voxel size) and cyst diameter was measured in T2WI 3D DRIVE images. When a cyst larger than 10–12 mm was detected, follow-up appointments were scheduled to ensure stability over time.

The number and dimensions (diameters) of the cysts are presented in **Table 1**. Both variables were converted from continuous to ordinal parameters, as described in the table footnote. Differences in the number and size of pineal cysts were compared between girls diagnosed with CPP and sex- and age-matched controls using Mann-Whitney non-parametric test.

**Table 1:** Number and size of pineal cysts in central precocious puberty and control groups (M±SD).

Variable	Girls with CPP	Controls
Cysts number *		
**0**	30/45 (67%)	28/47 (60%)
**1**	15/45 (33%)	19/47 (40%)
**2**	0 (0%)	0/47 (0%)
**3**	0 (0%)	0/47 (0%)
Cyst dimensions (diameter) °		
**0** (<5 mm)	30/45 (67%)	16/47 (80%)
**1** (5-10mm)	12/45 (27%)	4/47 (20%)
2 (>10 mm)	3/45 (6%)	0/47 (0 %)

CPP, central precocious puberty. * Number of cysts: 0: None; 1: 1 cyst; 2: 2 or 3 cysts; 3: more than 3 cysts. °Cyst dimensions: 0: diameter < 5mm; 1: 5 ≤ diameter ≤ 10 cyst; 2: diameter > 10 mm.

#### 2.3.2 Radiomic feature extraction

Radiomic features were computed with the open-source Python package PyRadiomics (39). Images were resampled to an isotropic voxel size of 1 × 1 × 1mm to minimize sampling-related variability. Only unprocessed image data were used, yielding 107 quantitative descriptors covering shape, size, intensity, and texture.

### 2.4 Models Development and Statistical Analysis

**Figure 1:**
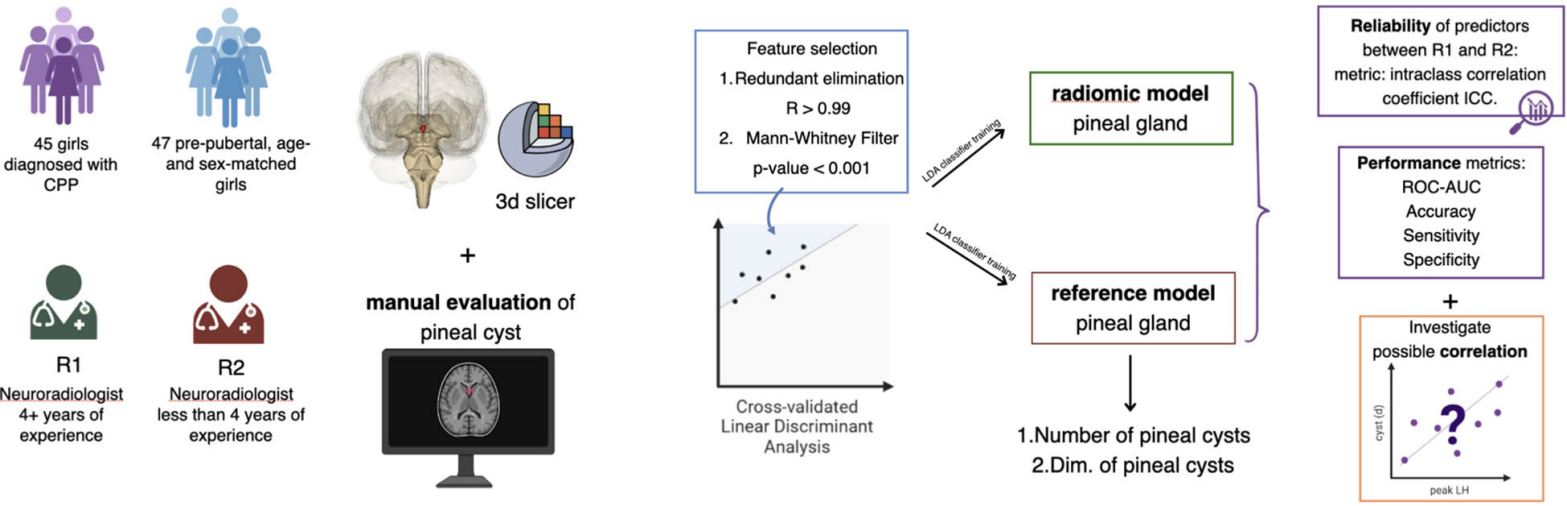
The methodological approach to radiomics analysis of the pineal gland in girls diagnosed with CPP and sex- and age-matched controls.

The methodological approach used for the models’ development and statistical analysis is summarized in **Figure 1**. Two models were set up using Monte Carlo Cross-Validation (MCCV, 100 stratified 80/20 splits): a radiomics-based classifier and a reference model based on cyst number and dimensions of the pineal gland. For the radiomic model, highly correlated features (Pearson *r* > 0.99) were excluded, and the Mann–Whitney test (*p*<0.001) was used to select the two features with higher discriminative capability based on cumulative score across MCCV iterations; no feature selection was instead applied to the reference model (number and size of cysts). Linear Discriminant Analysis (LDA) (40, 41) was trained and applied unchanged to validation set for both radiomic and reference models. Separate radiomic models were built for R1 and R2 segmentations, with the selected radiomic features by R1 also employed for R2 for robustness assessment: this allowed direct comparison of model variability and reliability with respect to reader expertise. Performance was evaluated using ROC (Receiver Operating Characteristic) and AUC (Area under the curve).

, accuracy, sensitivity, specificity, and geometric mean (G-Mean), averaged over MCCV iterations. Inter-reader consistency of feature extraction and pineal gland volume was assessed using intraclass correlation coefficients (ICCs) with a two-way random-effects model, interpreted as: <0.50, poor; 0.50–0.75, moderate; 0.75–0.90, good; >0.90, excellent (42). The correlation of both radiomic features and pineal cyst descriptors with basal/peak gonadotropin levels and estradiol was also explored. Spearman’s rho was used to assess correlations that involved radiomic features, whereas Kendall’s tau was applied for the pineal cyst descriptors. Analyses were performed in R (43) using *Caret* (44) and *MASS* (45).

## 3 Results

### 3.1 Clinical parameters

A total of 45 girls with CPP and 47 age-matched prepubertal controls were included in the radiomic analysis as previously described (29). The mean age of patients with CPP was 8.2 ± 0.6 years, and controls was 8.5 ± 1.2 years. BMI SDS of girls with CPP was -0.2 ± 1.0 and height SDS 1.0 ± 0.9. Other clinical, biochemical and pelvic US data of patients are reported in **Table 2**.

**Table 2:** Clinical, hormonal, and instrumental features of the girls diagnosed with central precocious puberty (M±SD).

Variable	Girls with CPP
	Mean ± SD
Chronological age, years	8.2 ± 0.6
Bone age, years	9.5 ± 1.2
Hormonal parameters	
Basal LH, mU/mL	1.6 ± 1.4
Basal FSH, mU/mL	4.7 ± 2.2
Peak LH, mU/mL	23.7 ± 16.0
Peak FSH, mU/mL	14.5 ± 4.6
Delta LH	22.2 ± 15.2
Delta FSH	9.6 ± 4.5
TSH, uUl/mL	2.1 ± 1.6
fT4, ng/dl	0.9 ± 0.1
Ultrasonographic parameters	
Right ovarian volume, mL	2.6 ± 1.2
Left ovarian volume, mL	2.7 ± 1.5
Uterine body length, mm	46.3 ± 8.1
Fundus/cervix ratio	1.5 ± 0.6

LH, luteinizing hormone; FSH, follicle-stimulating hormone; TSH, thyroid-stimulating hormone; fT4, free thyroxine. Peak hormonal values were acquired after GnRH stimulation, and delta values represented the differences between peak and basal LH and FSH values.

The presence of pineal cysts was an incidental finding in children with CPP. Comparison between pineal cysts in girls with diagnosed CPP and age- and sex-matched prepubertal controls is summarized in **Table 1**.

**Figure 2:**
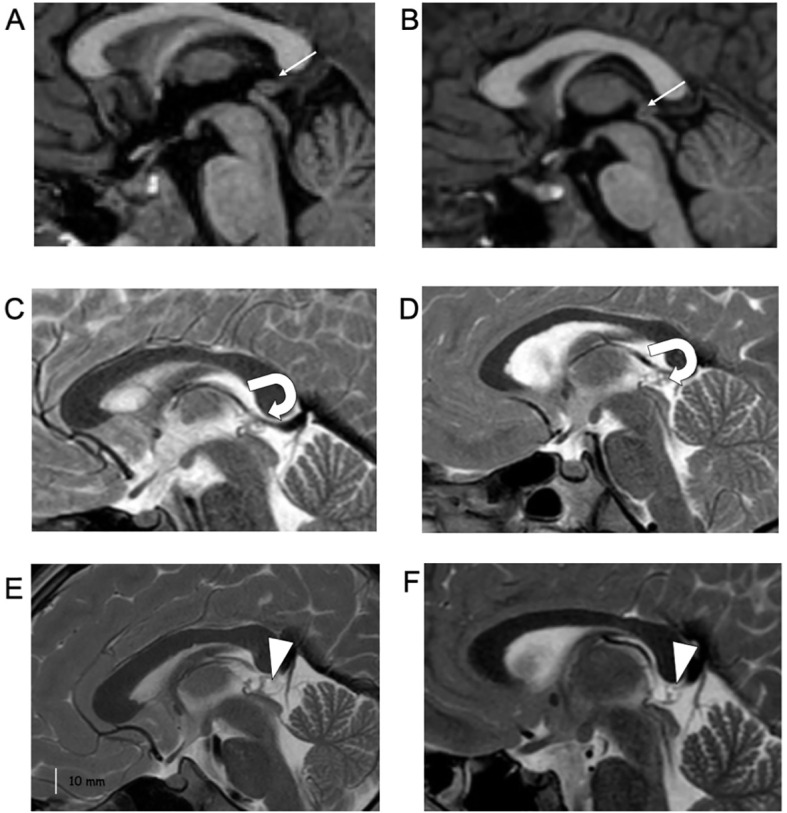
Comparative MRI characteristics of pineal gland in prepubertal controls and in girls with central precocious puberty. **(A, B)** Sagittal 3DMPRAGE images show the appearance of a normal pineal gland without pineal cyst in **(A)** prepubertal control and **(B)** CPP girl, respectively (arrows). **(C, D)** Sagittal T2 images show a microcystic pineal gland (curved arrow) in **(C)** prepubertal control and **(D)** CPP girls. It is characterized by small, well-defined hyperintense cystic components within the glandular parenchyma. **(E, F)** Macrocystic pineal gland in **(E)** prepubertal control and **(F)** CPP girl, respectively (arrow head). It is defined by larger fluid-filled cavities with thin smooth walls. A 10-mm scale bar is provided for reference.

Differences in the number and size of pineal cysts between CPP and control subjects were not statistically significant (p > 0.05) (**Figure 2**).

### 3.2 Radiomics of the pineal gland

**Figure 3:**
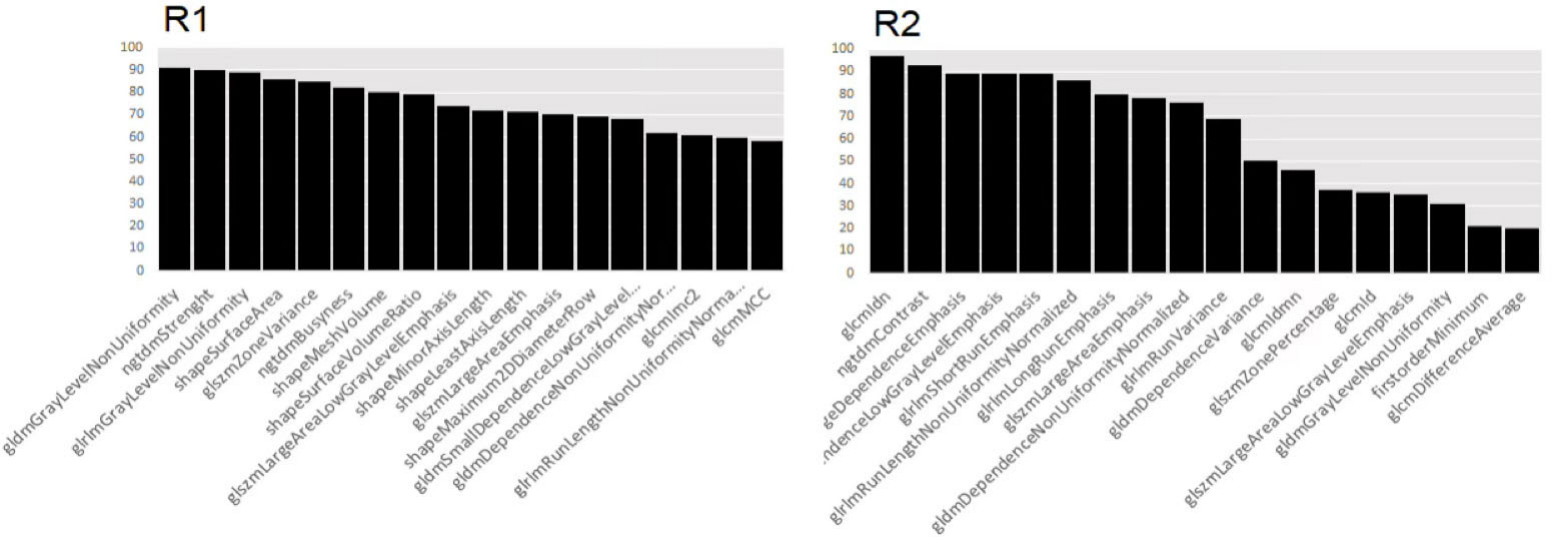
Cumulative scores of the top 18 radiomic features, calculated as the sum of scores obtained over 100 rounds. (R1) Feature scores derived from R1 segmentation and R2 feature scores, obtained from R2 segmentation.

The top-ranked RFs selected across 100 rounds of feature selection are shown in **Figure 3** for segmentations performed by Reader 1 (R1) and Reader 2 (R2), respectively. For R1, the highest-ranked features were *Gray Level Non-Uniformity*, which is a second-order texture feature from the GLDM matrix that accounts for the (non-)similarity of gray-level intensity values in the pineal gland and *NGTDM Strength*, which measures how structures appear clearly within the region of interest. For R2, *GLCM Inverse Difference Normalized (IDN)*, which quantifies image texture by measuring the local homogeneity of gray levels, and *Neighbourhood Grey Tone Difference Matrix Contrast*, which takes into account both the spatial intensity change and the dynamic range of gray levels inside the pineal volume. Moreover, the assessment of inter-reader agreement of radiomic features extracted from the pineal gland revealed poor reliability. The intraclass correlation coefficients (ICCs) for key radiomic features such as *gldm Gray Level Non Uniformity* and *ngtdm Strength* were 0.11 and 0.24, confirming poor agreement between readers.

**Figure 4:**
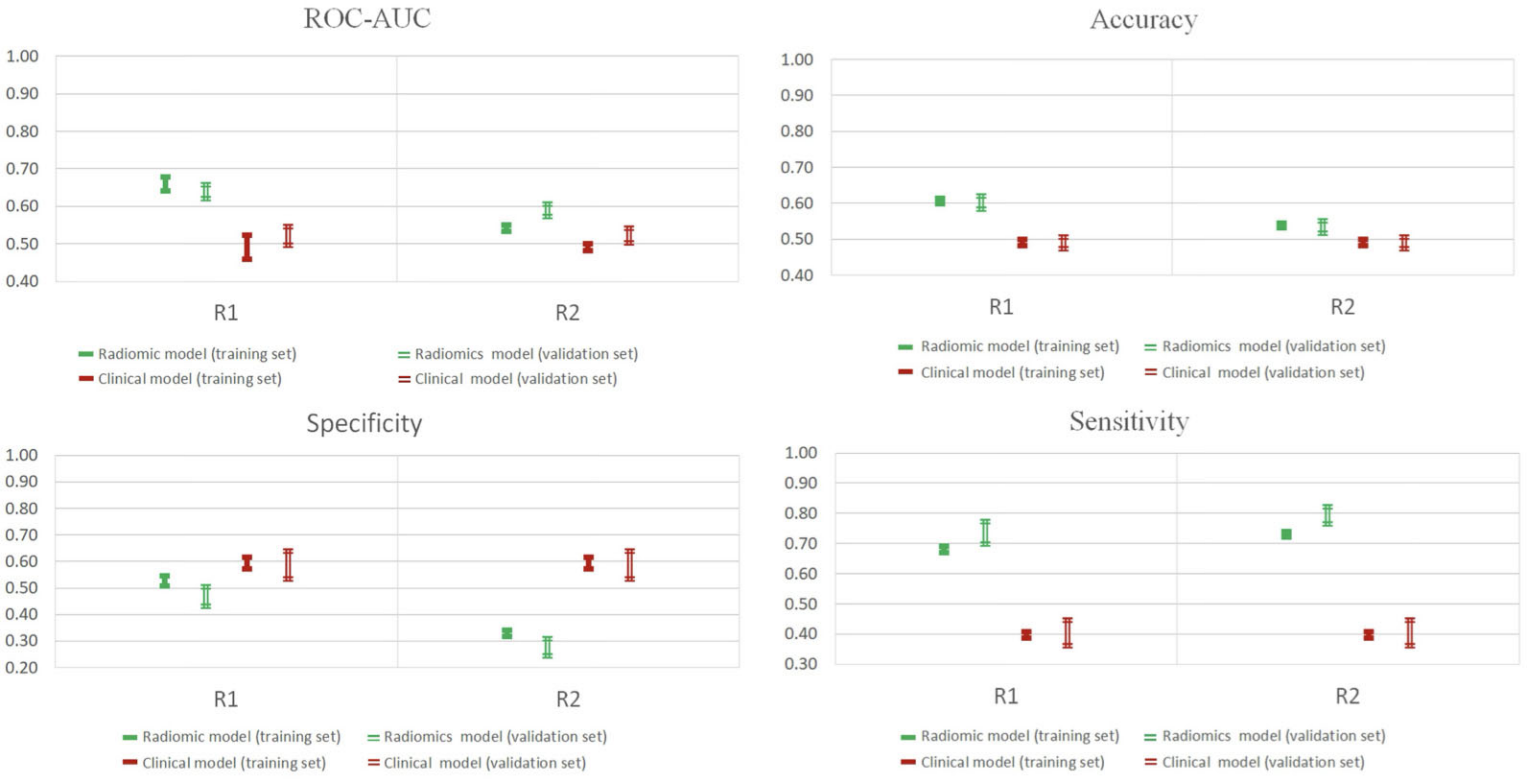
Model performances of radiomic and reference models. Error bars represent 95% confidence intervals. Single thick line: training set; Double thin lines: validation set; Green: radiomics; Red: number and diameter of pineal cysts.

In terms of predictive performance, radiomic features extracted from the pineal gland yielded only modest results. The radiomic models achieved ROC-AUC values of 0.64 (R1) and 0.59 (R2) on the validation set (**Figure 4**), while the clinical reference model reached 0.52. The accuracy, sensitivity, and specificity of all models were generally poor across training and validation. Radiomics showed relatively higher sensitivity but lower specificity compared with the reference model (based on the cyst number and size).

### 3.3 Correlation analyses of RFs with clinical, hormonal, and pelvic ultrasound data

Correlation analyses between radiomic features and potential confounders are summarized in **Table 3**. There was no significant correlation between the number or size of pineal cysts and basal or peak gonadotropin levels, and estradiol levels. Among clinical, hormonal and pelvic ultrasound data, a strong correlation was found between radiomic features and all hormonal parameters.

**Table 3:** Correlation analyses between hormonal levels in girls with CPP and number and size of pineal cysts and radiomic features.

Variables	Number of cysts °		Diameter of cysts °		gldm Gray Level Non Uniformity *		ngtdm Strength *	
	r	p	r	p	r	p	r	p
Basal LH, mU/mL	-0.12	0.31	-0.10	0.41	0.33	0.002	0.48	<0.001
Basal FSH, mU/mL	-0.11	0.38	-0.10	0.44	0.58	<0.001	0.60	<0.001
Peak LH, mU/mL	0.16	0.24	0.17	0.20	0.33	0.002	0.34	0.002
Peak FSH, mU/mL	0.17	0.21	0.17	0.19	0.59	<0.001	0.68	<0.001
Estradiol, pg/mL	-0.15	0.26	-0.17	0.20	-0.68	<0.001	-0.81	<0.001

LH, luteinizing hormone; FSH, follicle-stimulating hormone; r, correlation coefficient; p, p-value. ° The correlation was computed using Kendall Tau. * The correlation was computed by Spearman Rho.

## 4 Discussion

In this study, we applied a radiomic-based approach to brain MRI scans of the pineal gland in girls with central precocious puberty and sex- and age-matched controls. Despite promising findings from our previous investigation of the pituitary gland using similar methods (29), pineal gland radiomics did not provide sufficient radiomic features with discriminative power to support the diagnosis of CPP and, therefore, no significant changes associated with the onset of puberty were detected, although hormonal levels were associated with radiomic features (RFs).

To our knowledge, this is the first study investigating pineal gland radiomics in the context of CPP. Pineal cysts are commonly observed as incidental findings in pediatric MRI examinations. Although they are generally considered benign their notable prevalence among children has led to investigation into their possible impact on neuroendocrine function, particularly in the light of the pineal gland’s critical role in melatonin synthesis and secretion (17, 19, 22). A single-center Chinese study described an association between MRI-identified pineal cyst diameter (greater than 5mm) and an earlier onset of pubertal development (20). However, our findings showed that pineal cysts provided limited relevant diagnostic information in their radiomic profiles. Moreover, in this study, the size and number of cysts were not significantly different in CPP and controls, and showed no significant association with hormonal levels including basal and peak gonadotropin levels after GnRH stimulation at variance with the findings in the previous pituitary radiomic based model (29).

In detail, radiomic features, such as *Gray Level Non-Uniformity* and *Neighbourhood Grey Tone Difference Matrix Strength*, were regularly selected across models but their performance was limited. These two features showed a consistent moderate correlation with basal and peak LH and FSH levels, but they did not show any significant relationship with number or size of the cysts. These results suggest that specific radiomics features may reflect an internal microstructural variability of the pineal gland reflecting a normal anatomical variability of the pineal gland rather than processes related to puberty onset. Unlike our previous study, where pituitary radiomics showed high diagnostic accuracy (ROC-AUC = 0.81) (29), the pineal-based models showed low accuracy (ROC-AUC = 0.59–0.64), insufficient for clinical use. Importantly, the selected radiomic features were not influenced by anthropometric confounders, suggesting the reliability of segmentation and data extraction.

The overall radiomic features of pineal cysts were modest and the findings suggest that, unlike the pituitary gland, the pineal gland may not undergo consistent structural changes during the early stages of puberty, detectable by radiomics. The physiological variability of the pineal gland, including the frequent presence of cysts in healthy children, despite their number and size, underline the limited value of this region as a biomarker source of neuroendocrine regulation of pubertal timing.

One study reported a correlation between cyst size and markers of pubertal progression (20). This relationship was hypothesized to be associated with size of the cysts, and to subsequent compression of the region, and negative disruption of melatonin secretion or loss of melatonin’s inhibitory effect on the HPG axis (20), however, our current data would not support such a causal role. Moreover, other studies did not support these associations (22, 46). Therefore, in line with our results, pineal cysts may represent benign variability rather than having a significant meaning related to pubertal onset.

Finally, it must be considered that this study presents some limitations as the, images that were manually segmented may have contained manual errors. Improvement of standardization in image acquisition and feature extraction, possibly through automated or AI-assisted segmentation methods, could potentially improve the radiomic model. The small sample size also limits generalizability, and external validation in larger, independent cohorts is required. Lastly, the segmentation was performed by trained readers, however, differences in experience level may have introduced some variability in feature extraction.

In conclusion, the MRI-based radiomic features of the pineal gland in girls with CPP showed no significant differences when compared to prepubertal controls, and do not serve for diagnostic purposes. Furthermore, findings do not suggest any significant change in features with onset of puberty, at variance with the pituitary gland.

## Data Availability

The raw data supporting the conclusions of this article will be made available by the authors, without undue reservation.

## References

[1] Zevin EL Eugster EA . Central precocious puberty: a review of diagnosis, treatment, and outcomes. Lancet Child Adolesc Health. (2023) 7:886–96. doi: 10.1016/S2352-4642(23)00237-7. PMID: 37973253

[2] Pescovitz OH Eugster EA . Pediatric endocrinology, mechanisms, manifestations and management. Philadelphia: Lippincott Williams & Wilkins (2004) p. 63–79.

[3] Larsen PR Kronenberg HM Melmed S Polonsky KS . Williams Textbook of endocrinology, puberty ontogeny, neuroendocrinology, physiology and disorders. New York: Elsevier (2003).

[4] Carel JC Eugster EA Rogol A Ghizzoni L Palmert MR ESPE-LWPES GnRH Analogs Consensus Conference Group . Consensus statement on the use of gonadotropin-releasing hormone analogs in children. Pediatrics. (2009) 123:e752–62. doi: 10.1542/peds.2008-1783. PMID: 19332438

[5] Penco A Bossini B Giangreco M Vidonis V Vittori G Grassi N . Should pediatric endocrinologists consider more carefully when to perform a stimulation test? Front Endocrinol. (2021) 12:660692. doi: 10.3389/fendo.2021.660692. PMID: 33828534 PMC8021019

[6] Freire AV Escobar ME Gryngarten MG Arcari AJ Ballerini MG Bergadá I . High diagnostic accuracy of subcutaneous Triptorelin test compared with GnRH test for diagnosing central precocious puberty in girls. Clin Endocrinol. (2013) 78:398–404. doi: 10.1111/j.1365-2265.2012.04517.x. PMID: 22845185

[7] Chittwar S Shivprakash Ammini AC . Precocious puberty in girls. Indian J Endocrinol Metab. (2012) 16(Suppl 2):S188–91. doi: 10.4103/2230-8210.104036. PMID: 23565375 PMC3603023

[8] Latronico AC Brito VN Carel JC . Causes, diagnosis, and treatment of central precocious puberty. Lancet Diabetes Endocrinol. (2016) 4:265–74. doi: 10.1016/S2213-8587(15)00380-0. PMID: 26852255

[9] Stephen MD Zage PE Waguespack SG . Gonadotropin-dependent precocious puberty: neoplastic causes and endocrine considerations. Int J Pediatr Endocrinol. (2011) 2011:184502. doi: 10.1155/2011/184502. PMID: 21603196 PMC3212801

[10] Cantas-Orsdemir S Garb JL Allen HF . Prevalence of cranial MRI findings in girls with central precocious puberty: a systematic review and meta-analysis. J Pediatr Endocrinol Metab. (2018) 31:701–10. doi: 10.1515/jpem-2018-0052. PMID: 29902155

[11] Pedicelli S Alessio P Scirè G Cappa M Cianfarani S . Routine screening by brain magnetic resonance imaging is not indicated in every girl with onset of puberty between the ages of 6 and 8 years. J Clin Endocrinol Metab. (2014) 99:4455–61. doi: 10.1210/jc.2014-2702. PMID: 25238205

[12] Fava D Calandrino A Calevo MG Allegri AEM Napoli F Gastaldi R . Clinical, endocrine and neuroimaging findings in girls with central precocious puberty. J Clin Endocrinol Metab. (2022) 107:e4132–43. doi: 10.1210/clinem/dgac422. PMID: 35881919

[13] Robben SG Oostdijk W Drop SL Tanghe HL Vielvoye GJ Meradji M . Idiopathic isosexual central precocious puberty: magnetic resonance findings in 30 patients. Br J Radiol. (1995) 68:34–8. doi: 10.1259/0007-1285-68-805-34. PMID: 7881880

[14] Ilahi S Beriwal N Ilahi TB . Physiology, pineal gland. [Updated 2023 apr 24. In: StatPearls [Internet]. StatPearls Publishing, Treasure Island (FL (2025). Available online at: https://www.ncbi.nlm.nih.gov/books/NBK525955/ (Accessed August 19, 2025).

[15] Ebling FJ Foster DL . Pineal melatonin rhythms and the timing of puberty in mammals. Experientia. (1989) 45:946–54. doi: 10.1007/BF01953052. PMID: 2680575

[16] Patel S Rahmani B Gandhi J Seyam O Joshi G Reid I . Revisiting the pineal gland: a review of calcification, masses, precocious puberty, and melatonin functions. Int J Neurosci. (2020) 130:464–75. doi: 10.1080/00207454.2019.1692838. PMID: 31714865

[17] Andersen CC Kjær EKR Vase CB Mathiasen R Debes NM Jørgensen NR . Melatonin secretion across puberty: A systematic review and meta-analysis. Psychoneuroendocrinology. (2025) 173:107281. doi: 10.1016/j.psyneuen.2025.107281. PMID: 39823958

[18] Chen Z Si L Zhang X Wei C Shu W Wei M . Therapeutic effects of melatonin in female mice with central precocious puberty by regulating the hypothalamic Kiss-1/Kiss1R system. Behav Brain Res. (2024) 461:114783. doi: 10.1016/j.bbr.2023.114783. PMID: 38029845

[19] Yuan S Lin Y Zhao Y Du M Dong S Chen Y . Pineal cysts may promote pubertal development in girls with central precocious puberty: a single-center study from China. Front Endocrinol (Lausanne). (2024) 15:1323947. doi: 10.3389/fendo.2024.1323947. PMID: 38405141 PMC10885350

[20] Poza JJ Pujol M Ortega-Albás JJ Romero O en representación del Grupo de estudio de insomnio de la Sociedad Española de Sueño (SES) . Melatonin in sleep disorders. Melatonina en los trastornos de sueño. Neurologia (Engl Ed). (2022) 37:575–85. doi: 10.1016/j.nrl.2018.08.002. PMID: 30466801

[21] Cassio A Marescotti G Aversa T Salerno M Tornese G Stancampiano M . Central precocious puberty in Italian boys: data from a large nationwide cohort. J Clin Endocrinol Metab. (2024) 109:2061–70. doi: 10.1210/clinem/dgae035. PMID: 38308814 PMC11244209

[22] Filippo G Gaudino R Calcaterra V Villani A Bozzola E Bozzola M . Incidental pineal gland cyst in girls with early onset of puberty. Ital J Pediatr. (2022) 48:44. doi: 10.1186/s13052-022-01235-4. PMID: 35313951 PMC8935686

[23] Warsza B Due-Tønnessen P Due-Tønnessen P Pripp A Ringstad G Eide PK . Prevalence of pineal cysts in healthy individuals: Emphasis on size, morphology and pineal recess crowding. J Neurol Sci. (2023) 453:120801. doi: 10.1016/j.jns.2023.120801. PMID: 37741123

[24] Lambin P Rios-Velazquez E Leijenaar R Carvalho S van Stiphout RG Granton P . Radiomics: extracting more information from medical images using advanced feature analysis. Eur J Cancer. (2012) 48:441–6. doi: 10.1016/j.ejca.2011.11.036. PMID: 22257792 PMC4533986

[25] Lambin P Leijenaar RTH Deist TM Peerlings J de Jong EEC van Timmeren J . Radiomics: the bridge between medical imaging and personalized medicine. Nat Rev Clin Oncol. (2017) 14:749–62. doi: 10.1038/nrclinonc.2017.141. PMID: 28975929

[26] Senthilkumaran N Vaithegi S . Image segmentation by using thresholding techniques for medical images. Int J Comput Sci Eng. (2016) 6:1–13. doi: 10.5121/cseij.2016.6101

[27] Shen G Jin X Sun C Li Q . Artificial intelligence radiotherapy planning: automatic segmentation of human organs in ct images based on a modified convolutional neural network. Front Public Health. (2022) 10:813135. doi: 10.3389/fpubh.2022.813135. PMID: 35493368 PMC9051073

[28] van der Veen J Willems S Deschuymer S Robben D Crijns W Maes F . Benefits of deep learning for delineation of organs at risk in head and neck cancer. Radiother Oncol. (2019) 138:68–74. doi: 10.1016/j.radonc.2019.05.010. PMID: 31146073

[29] Maddalo M Petraroli M Ormitti F Fulgoni A Gnocchi M Masetti M . Magnetic resonance imaging -based radiomics of the pituitary gland is highly predictive of precocious puberty in girls: a pilot study. Front Endocrinol (Lausanne). (2025) 16:1496554. doi: 10.3389/fendo.2025.1496554. PMID: 39974824 PMC11835667

[30] Jiang H Shu Z Luo X Wu M Wang M Feng Q . Noninvasive radiomics-based method for evaluating idiopathic central precocious puberty in girls. J Int Med Res. (2021) 49:300060521991023. doi: 10.1177/0300060521991023. PMID: 33596690 PMC7897833

[31] Zou P Zhang L Zhang R Wang C Lin X Lai C . Development and validation of a combined MRI radiomics, imaging and clinical parameter-based machine learning model for identifying idiopathic central precocious puberty in girls. J Magn Reson Imaging. (2023) 58:1977–87. doi: 10.1002/jmri.28709. PMID: 36995000

[32] Marshall WA Tanner JM . Variations in pattern of pubertal changes in girls. Arch Dis Child. (1969) 44:291–303. doi: 10.1136/adc.44.235.291. PMID: 5785179 PMC2020314

[33] de Onis M Onyango AW Borghi E Siyam A Nishida C Siekmann J . Development of a WHO growth reference for school-aged children and adolescents. Bull World Health Organ. (2007) 85:660–7. doi: 10.2471/blt.07.043497. PMID: 18026621 PMC2636412

[34] Tanner JM Goldstein H Whitehouse RH . Standards for children’s height at ages 2–9 years allowing for heights of parents. Arch Dis Child. (1970) 45:755–62. doi: 10.1136/adc.45.244.755. PMID: 5491878 PMC1647404

[35] Cacciari E Milani S Balsamo A Spada E Bona G Cavallo L . Italian cross-sectional growth charts for height, weight and BMI (2 to 20 yr). J Endocrinol Invest. (2006) 29:581–93. doi: 10.1007/BF03344156. PMID: 16957405

[36] Bayer LM . Radiographic atlas of skeletal development of the hand and wrist: Second Edition. Calif Med. (1959) 91:53. doi: 10.1001/jama.1950.02910470084032. PMID: 33631893

[37] Talarico V Rodio MB Viscomi A Galea E Galati MC Raiola G . The role of pelvic ultrasound for the diagnosis and management of central precocious puberty: An update. Acta BioMed. (2021) 92:e2021480. doi: 10.23750/abm.v92i5.12295. PMID: 34738554 PMC8689311

[38] Fedorov A Beichel R Kalpathy-Cramer J Finet J Fillion-Robin JC Pujol S . 3D Slicer as an image computing platform for the quantitative imaging network. Magn Reson Imaging. (2012) 30:1323–41. doi: 10.1016/j.mri.2012.05.001. PMID: 22770690 PMC3466397

[39] van Griethuysen JJM Fedorov A Parmar C Hosny A Aucoin N Narayan V . Computational radiomics system to decode the radiographic phenotype. Cancer Res. (2017) 77:e104–7. doi: 10.1158/0008-5472.CAN-17-0339. PMID: 29092951 PMC5672828

[40] Izenman AJ . Linear discriminant analysis. In: Modern multivariate statistical techniques. Springer, New York (2013). doi: 10.1007/978-0-387-78189-1_8

[41] Dhamnetiya D Goel MK Jha RP Shalini S Bhattacharyya K . How to perform discriminant analysis in medical research? Explained with illustrations. J Lab Physicians. (2022) 14:511–20. doi: 10.1055/s-0042-1747675. PMID: 36531553 PMC9750738

[42] Koo TK Li MY . A guideline of selecting and reporting intraclass correlation coefficients for reliability research. J Chiropr Med. (2016) 15:155–63. doi: 10.1016/j.jcm.2016.02.012. PMID: 27330520 PMC4913118

[43] R Core Team . R: A language and environment for statistical computing. Vienna, Austria: R Foundation for Statistical Computing Vienna (2021). Available online at: https://www.R-project.org/ (Accessed March 05, 2025).

[44] Kuhn M . Building predictive models in r using the caret package. J Stat Software. (2008) 28:1–26. doi: 10.18637/jss.v028.i05

[45] Venables WN Ripley BD . Modern Applied Statistics with S, Fourth edition. New York: Springer (2002). Available online at: https://www.stats.ox.ac.uk/pub/MASS4/ (Accessed July 07, 2025).

[46] Çıracı S Polat R . Pineal cysts in children with precocious puberty: Is it a coincidental finding? Sakarya Tıp Dergisi. (2022) 12:315–21. doi: 10.31832/smj.1060348

